# Designing and Implementing a Competency-Based Training Program for Anesthesiology Residents at the University of Ottawa

**DOI:** 10.1155/2015/713038

**Published:** 2015-12-21

**Authors:** Emma J. Stodel, Anna Wyand, Simone Crooks, Stéphane Moffett, Michelle Chiu, Christopher C. C. Hudson

**Affiliations:** ^1^Learning 4 Excellence, 749 Long Point Circle, Ottawa, ON, Canada K1T 4H5; ^2^Department of Anesthesiology, The Ottawa Hospital, 1053 Carling Avenue, Ottawa, ON, Canada K1Y 4E9; ^3^University of Ottawa Heart Institute, 40 Ruskin Street, Ottawa, ON, Canada K1Y 4W7

## Abstract

Competency-based medical education is gaining traction as a solution to address the challenges associated with the current time-based models of physician training. Competency-based medical education is an outcomes-based approach that involves identifying the abilities required of physicians and then designing the curriculum to support the achievement and assessment of these competencies. This paradigm defies the assumption that competence is achieved based on time spent on rotations and instead requires residents to demonstrate competence. The Royal College of Physicians and Surgeons of Canada (RCPSC) has launched Competence by Design (CBD), a competency-based approach for residency training and specialty practice. The first residents to be trained within this model will be those in medical oncology and otolaryngology-head and neck surgery in July, 2016. However, with approval from the RCPSC, the Department of Anesthesiology, University of Ottawa, launched an innovative competency-based residency training program July 1, 2015. The purpose of this paper is to provide an overview of the program and offer a blueprint for other programs planning similar curricular reform. The program is structured according to the RCPSC CBD stages and addresses all CanMEDS roles. While our program retains some aspects of the traditional design, we have made many transformational changes.

## 1. Introduction

Around the world, medical education is undergoing a major transformation. Calls for reform in how physicians are trained are longstanding [1]. In many of the current systems, programs define the successful completion of training based on the length of time a trainee is in a program, assuming that trainees will develop the required competencies to practice after a predetermined amount of time. However, factors such as the restrictions around resident duty hours [2], pressure to reduce costs associated with resident training [3], and the need for improved accountability for patient safety [4] have led many countries to consider a competency-based approach to medical education. Competency-based medical education is an outcomes-based approach that involves identifying the abilities required of the physician and then designing the curriculum to support the achievement of these predefined competencies. This paradigm defies the assumption that competence to practice as a fully rounded physician is achieved based on time spent on rotations, that is, through an on-the-job training and/or apprenticeship model [5], and instead requires residents to demonstrate the competencies deemed necessary for patient care. For some trainees, this may require more time than the typical programs, while others may be able to accelerate their training and enter the workforce earlier and/or engage in further specialized training.

By definition, competency-based medical education needs a robust and multifaceted assessment system. Multiple data points are needed to make judgments about residents' competence. Assessments need to occur in a combination of classroom, clinical, and simulated environments and be criterion based. Frequent assessment enables the provision of high quality feedback to guide development of competence and facilitates the early identification of both residents in difficulty and those who may benefit from focused training for rapid advancement.

The Royal College of Physicians and Surgeons of Canada (RCPSC) has launched a multiyear initiative to implement a competency-based medical education approach to residency training in Canada called Competence by Design (CBD) [6]. The RCPSC's CBD program focuses on physicians' lifelong learning from the time they graduate from medical school to retirement. The CBD Competence Continuum [7] breaks down physician education into a series of stages: Transition to Discipline, Foundations of Discipline, Core of Discipline, Transition to Practice, Continuing Professional Development, and Transition out of Professional Practice.

Within each stage of resident training (from Transition to Discipline to Transition to Practice) there are a series of Entrustable Professional Activities (EPAs) and milestones. According to the RCPSC, an EPA “is a task in the clinical setting that may be delegated to a resident by their supervisor once sufficient competence has been demonstrated” [8]. Each EPA typically includes several milestones. Milestones are “observable marker[s] of an individual's ability along a developmental continuum” [8]. So, while an EPA may be considered an end task or activity (e.g., management of cardiac surgery using cardiopulmonary bypass), the milestones are the abilities of the individual needed to be able to complete the task (e.g., place and interpret a pulmonary artery catheter). The expectation from the RCPSC is that milestones will be used to design curriculum and then EPAs used to assess resident competence [8]. If a resident is struggling with an EPA, the milestones can be examined to see which individual abilities the resident is lacking and thus determine where focused training and support are needed.

The first two specialties to implement the RCPSC's CBD program will be medical oncology and otolaryngology-head and neck surgery in 2016. However, following the successful application for a Fundamental Innovations in Residency Education (FIRE) project (http://www.royalcollege.ca/portal/page/portal/rc/resources/publications/oe_news/vol6_1/fire_project) from the RCPSC, the Department of Anesthesiology, University of Ottawa, launched a competency-based residency training program on July 1, 2015, two years ahead of the RCPSC schedule for national implementation of CBD for anesthesiology. This is the second program in Canada to make this transformational change (following the orthopedic surgery residency program in Toronto). The purpose of this paper is to provide an overview of the University of Ottawa anesthesiology competency-based residency program and to offer a blueprint for other programs that plan to reform their training to meet 21st century healthcare needs.

## 2. Methods

While the entire faculty of the Department of Anesthesiology is involved in this initiative, a smaller Education Design Steering Group guided the conceptual design of the program and submitted the FIRE project application to the RCPSC. The FIRE proposal described the EPAs and milestones for the program based on the National Curriculum for Canadian Anesthesia Residency [9], as well as innovative and nontraditional approaches for teaching and assessment designed to support and evaluate residents' achievement of the EPAs and milestones. The Education Design Steering Group defined EPAs that are to be achieved by the end of the program, with milestones for each stage of training. This is in contrast to the RCPSC that has defined EPAs for each stage of training. The proposed program also eliminates curricula that do not contribute to the achievement of the defined EPAs and milestones, thus maximizing clinical exposure to include only those critical elements for the practice of anesthesia. As a result, residents who demonstrate all required competencies without the need for remediation will be able to complete their training in four years instead of the current five-year minimum as per the RCPSC specialty training requirements in anesthesiology [10]. With the FIRE project approved by the RCPSC, the Department of Anesthesiology started work on further developing the new curriculum. The objectives for the new competency-based program were as follows:

1. To adopt an outcomes-based approach to program and curriculum design by
  - using an evidence-based approach to develop EPAs and milestones to shape a modular curriculum;
  - leveraging faculty expertise in the development of EPAs and milestones.
2. To develop a curriculum that cultivates and assesses competency in all seven CanMEDS roles equally [11].
3. To use a learner-centered approach that emphasizes active learning processes and recognizes that trainees learn differently and at different speeds.
4. To build in opportunities to encourage resident self-reflection as a means of learning and self-assessment [12].
5. To create a modular-based spiral curriculum that revisits and reinforces core and subspecialty competencies of anesthesia.
6. To develop a robust formative and summative assessment approach.
7. To control progression through the program based on achievement of milestones through formative and summative assessments and thus identify residents in difficulty early and establish appropriate supports.
8. To establish a faculty development program to support the implementation of the new program.
9. To support change management as appropriate for faculty and residents.
10. To disseminate best practices for teaching and assessment nationally and internationally.
11. To engage in continuous evaluation of the program both internally and externally during both the development and implementation stages.
12. To identify economies of scale and economies of scope as appropriate in the development of learning and assessment strategies.

## 3. Program Description

The new competency-based anesthesiology residency program at the University of Ottawa is structured according to the RCPSC CBD stages [7]: Transition to Discipline, Foundations of Discipline, Core of Discipline, and Transition to Practice. The instructional design of the residency training stages is geared towards the assessment and achievement of the EPAs and milestones and is described in the following sections.

### 3.1. Transition to Discipline

Transition to Discipline comprises one four-week rotation (one block) on general adult anesthesia that serves as an introduction and orientation to the specialty, the university, and the Department of Anesthesiology. Further, it provides an opportunity to assess residents' clinical skills and knowledge and to orient them to on-call activities through a “buddy call” system. “Buddy call” involves pairing the novice resident with a more senior resident deemed competent for independent call. Two faculty member leads (Anna Wyand and Stéphane Moffett) were recruited and, in consultation with an educational expert (Emma J. Stodel), were responsible for designing the curriculum and assessment approaches for this stage of training.

Residents are provided with an orientation package and required reading two weeks prior to the start of the program. Transition to Discipline starts with an orientation day where residents are oriented to both the physical locations within which they will practice and logistical resources they will require during residency. They are introduced to the process for setting up invasive hemodynamic monitors and point-of-care testing systems.

Following orientation, residents engage in daily elective and on-call clinical anesthesia. Clinical exposure is standardized for all residents. Residents are responsible for completing a number of tasks described on the Anesthesia Resident Checklist. This checklist serves as a guide to continue to orient residents to the locations, equipment, and on-call responsibilities they will be working towards achieving over the course of Transition to Discipline. Residents are also required to seek feedback on seven supervised clinical skills including endotracheal intubation, intravenous cannulation, and postanesthesia care unit (PACU) handover to a registered nurse. A mandatory lecture series provides introductions to the CanMEDS framework [13], the preoperative and intraoperative patient management software, and how to safely check the anesthesia machines.

During Transition to Discipline, residents are assessed on their clinical competence using the Clinical Case Assessment Tool (CCAT), an online daily assessment tool used to assess competence in all CanMEDS roles (see full description in Section 3.6). Knowledge is assessed using the Anesthesia Knowledge Test 1 (AKT-1 pretest) (http://www.metricsinc.org/akt-testing.html), which assesses their incoming knowledge, and the AKT-1 posttest administered one month later (at the start of Foundations of Discipline). Residents who fail to complete the educational requirements within the four weeks of Transition to Discipline must remediate during the following two weeks in order to be permitted to continue in the Foundations of Discipline stage.

### 3.2. Foundations of Discipline

Foundations of Discipline comprises a 12-week (three-block) Boot Camp followed by a 12-week (three-block) clinical component. One faculty member was recruited to design Boot Camp (Simone Crooks) and two were recruited to design the clinical component of the stage (Anna Wyand and Stéphane Moffett).

The Foundations Boot Camp is designed to accelerate the development of junior residents' technical and nontechnical skills in a safe learning environment so they are at a relatively advanced level when in the clinical environment providing patient care. The Boot Camp approach has gained a lot of attention recently, especially in the surgical fields [14–18]. Boot Camps enable residents to develop clinical skills in a lower stress environment with less time pressure than they would face in a clinical setting, and they let residents self-reflect and receive feedback in a nonthreatening environment. Further, deficiencies in skills and knowledge can be identified early and remediated.

A typical Boot Camp week in our new competency-based anesthesiology program comprises two days of clinical work; two days of instructional sessions that include partial task training, workshops, lectures, and problem-based learning; and one day of high fidelity simulation. Residents complete 18 high fidelity simulation scenarios geared towards a junior level of training. Simulation sessions are formatively assessed and residents engage in a formal debriefing with faculty simulation instructors. Lectures in Boot Camp cover both clinical topics and the intrinsic CanMEDS roles. In addition, residents complete the following courses: Advanced Trauma Life Support (ATLS), Advanced Cardiac Life Support (ACLS), and Acute Critical Events Simulation (ACES) (an interactive course on the acute resuscitation of critically ill patients and the management of crisis situations).

Following successful completion of Boot Camp, residents progress to the clinical component of Foundations. This provides them with the opportunity to consolidate their knowledge of basic anesthesiology and refine their technical skills in the clinical setting. Over the course of the 12-week clinical component, the residents are scheduled in the main operating room, the obstetrical operating room, and the preoperative assessment unit and acute pain service. Similar to Transition to Discipline, residents are given a checklist of tasks they must complete. During this part of their training, residents transition from “buddy call” to solo call responsibilities.

Assessment is multifaceted. Formative assessment of simulated crisis management in Boot Camp involves the use of checklists following partial task training workshops, online modules, and postcrisis management reflective exercises. As with Transition to Discipline, clinical competence is assessed using the CCAT. In addition, attending staff use checklists to assess the residents' skills with epidural and spinal techniques. Summative assessment includes the AKT-6 six months into the program and an eight-station Objective Structured Clinical Examination (OSCE) designed to assess the Foundations of Discipline milestones. Two expert raters judge residents' performance on the OSCE at each station using modified Delphi-derived checklists and the Managing Emergencies in Pediatric Anesthesia Global Rating Scale (MEPA GRS) [19]. Residents who fail to meet the milestones of Foundations of Discipline do not progress to the next stage of training.

### 3.3. Core of Discipline

Core of Discipline comprises 13 subspecialty-specific modules based on the National Curriculum for Canadian Anesthesia Residency [9]: acute pain, airway, cardiac anesthesia, chronic pain, clinical pharmacology and complex surgery, neuroanesthesia, obstetrical anesthesia, pediatric anesthesia, perioperative medicine, regional anesthesia, remote location anesthesia, thoracic anesthesia, and vascular anesthesia. The Core of Discipline stage is divided into Core I and Core II, equivalent to junior and senior residency, respectively, over 40 four-week blocks. Core II revisits and reinforces more advanced core and subspecialty competencies of anesthesia first addressed in Core I. Subspecialty experts were recruited as Module Leads and were responsible for designing the curriculum and assessment approaches for their module with the support of an educational expert (Emma J. Stodel).

Each module has predefined educational requirements designed to help residents achieve the defined milestones and EPAs. Educational activities include presenting rounds; partial task training; participating in required clinical case experiences, procedures, and techniques; preparing for and writing case summaries; and completing learning cases specific to each module. The learning cases are designed to replace the RCPSC mandated core academic half-days, which usually comprise expert-led lectures that are typically not synchronized with relevant clinical experiences. Our learning cases are linked to the module the resident is in so residents learn topics relevant to their current clinical context. For example, if they are in the pediatrics module they only complete pediatric learning cases.

The learning cases are based on the format of the RCPSC oral examinations. Residents are provided with a case scenario followed by questions to direct their learning, as well as selected resources (e.g., journal articles, textbook chapters, videos, and learning activities). Residents are expected to spend one to one-and-a-half hours engaged in self-directed learning for each case. They then meet with a staff anesthesiologist to discuss the case and are assessed against the expected level of competence defined in the case. This design enables residents to complete the cases when and where they choose and spend as long on each case as required. By reviewing the topic before interacting with staff, the staff-resident discussions can be at a more advanced level, mirroring a “flipped classroom” [20]. The content of the learning cases is resource based and curated by subject matter experts, thus minimizing demands on staff time for case creation. Staff have also shown interest in using these cases for their own Continuing Professional Development.

Learning cases are delivered through a custom-built electronic system that stores and manages access to cases, tracks completion, and documents assessment. Permission settings restrict access to the staff versions of the cases that list the critical features that residents need to cover to demonstrate understanding and determine who can assess residents on the cases. Data from this system will be automatically fed into in-training evaluation reports (ITERs) so evaluators know whether module requirements have been satisfied, as well as to a central dashboard that will provide a summary of resident progress. An overview of the learning case platform can be found at https://www.youtube.com/watch?v=OLirP-QjFQA.

Simulation-based education continues through this and the next stage of training with a four-year rotation of core topics via the Global Simulation Curriculum. Delivery of this curriculum is coordinated by the Department of Anesthesiology Simulation Director (Michelle Chiu). In Core of Discipline and Transition to Practice, residents complete 33 high fidelity simulation scenarios of higher complexity than those experienced in Foundations of Discipline. The scenarios are designed to reflect rare, but important, clinical situations that residents may never be exposed to in residency and/or clinical situations that are critical to competency as an anesthesiologist. Fifty percent of all simulation scenarios are interprofessional and this proportion is growing. All the simulation scenarios focus on the intrinsic CanMEDS roles, as well as the role of Medical Expert. Objectives come from the Association of Canadian University Departments of Anesthesia (ACUDA) National Curriculum for Canadian Anesthesia Residency document [9]. During Core of Discipline and Transition to Practice, residents also participate in several interdisciplinary high fidelity simulation sessions with other specialties, such as obstetrics/gynecology, surgery, and emergency medicine.

Module Leads determined how competency is assessed in their module. Assessment tools include the CCAT, checklists, multiple-choice examinations, short answer questions, and case summaries where residents are required to reflect on the case and make links to best practices and the literature. Residents are formatively assessed following simulation scenarios and summatively assessed against the ACLS and Canadian National Anesthesiology Simulation Curriculum (CanNASC) Simulation Milestones. Assessment of a resident's management of a perioperative cardiac arrest (ACLS Simulation Milestone) occurs midway through the training program using high fidelity simulation. The CanNASC Simulation Milestone is assessed at the end of Core of Discipline using nationally developed and standardized high fidelity simulation scenarios and accompanying rubrics.

If residents fail to meet the required competencies by the end of Core I and Core II they complete supplemental blocks focusing on remediating gaps in their knowledge and skills before progressing to the next stage of training (i.e., Core II or Transition to Practice).

### 3.4. Transition to Practice

Transition to Practice is the final stage of CBD and is designed to prepare residents for autonomous practice. Residents engage in independent practice across a wide scope of subspecialties, working, in effect, as junior staff members. Staff anesthesiologists are immediately available to assess, guide reflection, and provide support as needed. As in the earlier stages of training, residents are required to complete a checklist of tasks and complete simulation scenarios and continue to be assessed using the CCAT. Further, they are encouraged to complete oral examination questions daily. The RCPSC specialty examination is a milestone in the Transition to Practice stage and one of a number of requirements for certification. Passing the examination will not lead to certification. The RCPSC will only grant certification when the resident has successfully completed the Transition to Practice stage and received sign-off (using the MAINPORT ePortfolio) from the Program Director. Allowing the resident to practice autonomously while continuing to evaluate competence ensures that we prepare physicians for practice in a way that is fundamentally oriented to graduate success and patient safety.

### 3.5. Intrinsic CanMEDS Roles

The teaching and assessment of the intrinsic CanMEDS roles (Communicator, Collaborator, Leader, Health Advocate, Scholar, and Professional) occurs throughout the program. Faculty member Role Leads are assigned to each role to support the teaching and assessment of the role. The roles are first introduced during Transition to Discipline through a session on the CanMEDS framework [13]. In Foundations of Discipline, each role is addressed through an interactive session involving small group discussions, case-based learning, educational “games” (e.g., scratch card quizzes), role playing, reflections, online modules, self-assessments, and small assignments. For example, for the Collaborator role, residents interview other members of the healthcare team to develop an understanding of their role, responsibilities, skill set, and training. Throughout Core of Discipline and Transition to Practice there are three sessions a year, organized by themes (e.g., ethics in anesthesia practice, safety, and quality improvement in anesthesiology practice), which specifically address the intrinsic roles using similar approaches to those described for Foundations.

The CCAT is used on a daily basis to document competence in the intrinsic roles. Residents are responsible for soliciting feedback on all of the CanMEDS competencies over the course of the block. A counter displays the number of CCATs completed for each role during the current block. Residents and staff are responsible for ensuring that all roles have been assessed and documented before the end of the block.

Competency within the intrinsic CanMEDS roles is further developed through an online coaching platform by encouraging reflection on practice in each of the roles. Residents are expected to post at least one reflection related to each role each year of their training (i.e., at least six posts a year) but are encouraged to post more. Every two months, residents receive an automated email prompting them to reflect on a specific role and post their reflection on the coaching platform within two weeks. Guidelines are provided to assist residents as they reflect. Role Leads support the residents through online coaching to deepen their reflections and develop competence in the intrinsic CanMEDS roles.

### 3.6. Clinical Case Assessment Tool (CCAT)

As multiple data points are needed to make judgments about residents' competence, frequent resident assessments are essential to the program's success. We needed a new assessment tool that would be intuitive to use, engage both staff and residents in the assessment process, and assess competence in all CanMEDS roles. We therefore developed the CCAT; an online resident-driven assessment tool that capitalizes on faculty's ability to judge competent clinical performance. The CCAT is designed to increase both face-to-face feedback to residents and resident self-assessment. Residents are required to complete at least 12 CCATs every four-week block throughout the program.

The CCAT was adapted from the validated Ottawa Surgical Competency Operating Room Evaluation (O-SCORE) [21] and is completed electronically on a computer or tablet. The CCAT is initiated by the resident who is required to reflect on his/her performance and assess him/herself prior to receiving assessment and face-to-face feedback from the supervising staff anesthesiologist. Residents enter identifying data about the case and then reflect on what they did well, what they would do differently next time, and the next steps in their learning plan. Responses are organized by CanMEDS role. The resident's self-assessment is then shared with the staff who assesses resident performance on a three-item, five-response option behaviorally anchored scale ranging from “Staff had to do” to “Staff did not need to be there” and documents their assessment of what was done well, what needs to be improved, and next steps for learning. Staff use the resident's device to do this, thus promoting timely face-to-face feedback.

The electronic format of the tool allows data to be easily analyzed and interpreted for evaluation, learning, research, and quality improvement purposes. A video tutorial demonstrating the CCAT can be found at https://www.youtube.com/watch?v=k6-Gj8ZDjhs.

## 4. Conclusion

A paradigm shift is occurring in the way in which physicians are trained. Globally, regulatory bodies are considering a shift to competency-based medical education as an answer to the mounting barriers and challenges of time-based models of training. The RCPSC is in the early stages of implementing the CBD program to all specialty training, with the first programs starting July 1, 2016. The Department of Anesthesiology at the University of Ottawa was granted permission from the RCPSC to pilot a competency-based medical education anesthesiology program two years ahead of the rest of the country. While our program retains some aspects of the traditional design, we made many key changes. First, our program offers the potential for residents to complete the program in four years instead of five if they demonstrate the required competence. The possible reduction in training time represents potential savings to the healthcare system through the provincial government subsidy for residency training. Second, the replacement of academic half-days with learning cases ensures that residents' learning outside the clinical setting is coordinated with their clinical experience and is focused on the subspecialty of the module they are in. Third, assessment is more robust and more frequent. This enables early identification of residents' strengths and weaknesses so remediation and/or tailored learning experiences can be implemented to accelerate learning and progression to new skills and knowledge. Further, the electronic nature of many of the assessments used facilitates tracking and reporting. Fourth, our program clearly defines the educational requirements for each stage of training and module. These must be completed before residents can progress to the next stage of training. Lastly, we have introduced a new, comprehensive curriculum for the teaching and assessment of the intrinsic CanMEDS roles.

This paper provides a comprehensive overview of our implementation of a competency-based approach to resident education and offers a blueprint for other programs planning a similar curricular reform. An in-depth evaluation of the program is underway, guided by a logic model and evaluation framework. Findings from the evaluation will be disseminated as they become available.
